# Is “Appearing Chronically Ill” a Sign of Poor Health? A Study of Diagnostic Accuracy

**DOI:** 10.1371/journal.pone.0079934

**Published:** 2013-11-27

**Authors:** Shail Rawal, Mina Atia, Rosane Nisenbaum, Dwayne E. Paré, Steve Joorden, Stephen W. Hwang

**Affiliations:** 1 Department of Medicine, University of Toronto, Toronto, Ontario, Canada; 2 Centre for Research on Inner City Health, The Keenan Research Centre in the Li Ka Shing Knowledge Institute of St. Michael’s Hospital, Toronto, Ontario, Canada; 3 Centre for Computational Cognitive Neuroscience, Department of Psychology, University of Toronto Scarborough, Toronto, Ontario, Canada; Universidad Peruana Cayetano Heredia, Peru

## Abstract

**Objective:**

To determine the sensitivity and specificity of a physician’s assessment that a patient “appears chronically ill” for the detection of poor health status.

**Methods:**

The health status of 126 adult outpatients was determined using the 12-Item Short Form Health Survey (SF-12). Physician participants (n = 111 residents and faculty) viewed photographs of each patient participant and assessed whether or not the patient appeared chronically ill. For the entire group of physicians, the median sensitivity and specificity of “appearing chronically ill” for the detection of poor health status (defined as SF-12 physical health score below age group norms by at least 1 SD) were calculated. The study took place from February 2009 to January 2011.

**Results:**

Forty-two participants (33%) had an SF-12 physical health score ≥1 SD below age group norms, and 22 (18%) had a score ≥2 SD below age group norms. When poor health status was defined as an SF-12 physical score ≥1 SD below age group norms, the median sensitivity was 38.1% (IQR 28.6–47.6%), specificity 78.6% (IQR 69.0–84.0%), positive likelihood ratio 1.64 (IQR 1.42–2.15), and negative likelihood ratio 0.82 (IQR 0.74–0.87). For an SF-12 physical score ≥2 SD below age group norms, the median sensitivity was 45.5% (IQR 36.4–54.5%), specificity 76.9% (IQR 66.3–83.7%), positive likelihood ratio 1.77 (IQR 1.49–2.25), and negative likelihood ratio 0.75 (IQR 0.66–0.86).

**Conclusions:**

Our study suggests that a physician’s assessment that a patient “appears chronically ill” has poor sensitivity and modest specificity for the detection of poor health status in adult outpatients. The associated likelihood ratios indicate that this assessment may have limited diagnostic value.

## Introduction

Physicians are taught to begin the physical examination with a general survey that includes an assessment of whether the patient appears acutely or chronically ill [1], [2]. In addition, a description of the patient’s apparent health status is a common component of formal case presentations [3]–[5]. This practice is grounded in the assumption that a patient’s apparent state of health correlates with their *actual* state of health, and that patients who appear chronically ill are more likely to suffer from one or more chronic illnesses. Physicians in training are also encouraged to hone their skills in the assessment of apparent health status as they are taught that this assessment can inform clinical decision-making. However, the evidence for this component of the physical examination is limited, and there exists little consensus as to what defines “appearing chronically ill”. Rather, this assessment is left to the individual physician. Thus, the goal of this study is to determine whether a physician’s assessment that a patient “appears chronically ill” is a sensitive or specific sign for the detection of poor health, as determined using the 12-item Short Form Health Survey (SF-12) as the reference standard.

## Materials and Methods

### Patient Participants

This study used patient data collected for a previous study of whether looking older than one’s actual age is a sign of poor health [6]. A total of 126 adult outpatients were recruited between February and April 2009 from four primary care clinics and one general internal medicine clinic affiliated with an academic teaching hospital in a large urban centre. A convenience sample of patients was generated by study investigators who approached patients in clinic waiting areas to determine their interest in participating in the study. Patients were excluded if they could not speak or read English. After informed consent was obtained, participants were asked to complete a survey to collect basic demographic information. A digital photograph was then taken of each patient participant, showing a frontal view of the individual’s face with a neutral facial expression.

The health status of each patient participant was determined using the self-administered version of the SF-12. Although the SF-12 was originally developed for use in large population health surveys, its brevity has led to its expanded use in the assessment of overall generic health status in smaller populations and in individuals. The SF-12 generates a physical composite score and mental composite score that are well-validated measures of general physical health and general mental health respectively [7]. Higher composite scores indicate better health status and are normalized to a mean of 50 and a standard deviation (SD) of 10 in the general United States adult population. We defined poor health status as the presence of a physical health score ≥1.0 SD (≥10 points) and ≥2.0 SD (≥20 points) below the patient’s age-group norm, as scores in this range are typically found in individuals with serious health impairments [7].

### Physician Participants

Physician participants were recruited by an email invitation sent to all internal medicine residents in the first three years of postgraduate training, and all general internal medicine faculty physicians at the University of Toronto. Physician participants were directed to a web-based computer program and asked to rate individual photographs of each of the 126 patient participants. Physician participants were blinded to the specific objectives of the study and to the patients’ SF-12 scores. The following instructions were provided with each image: “This patient is [age] years old. Do you think this patient looks chronically ill?” To reduce systematic bias due to rater fatigue, the computer program randomized the order in which the photographs were presented so that each physician viewed the photographs in a different order. There was no time limit to view each photo, but physicians were encouraged to complete the survey in a single sitting. Physicians were not given information on whether the patient participants were inpatients or outpatients. Upon completion, physicians were asked to disclose whether they were a resident or faculty member. Data from physicians was collected from December 2010 to January 2011. Examples of actual images presented to physicians are shown in Figure S1, Figure S2, Figure S3, Figure S4.

All patient and physician participants gave written informed consent for participating in the study. Each patient participant received a small honorarium and two physician participants were randomly selected to receive an honorarium. This study was approved by the Research Ethics Board of St. Michael’s Hospital, Toronto, Canada.

### Statistical Analyses

In this descriptive study, sample size was not computed. The study utilized patient data from a previous study, and the authors targeted a sample size large enough to yield precise estimates of the sensitivity and specificity of a physician’s assessment that a patient “appears chronically ill”. For each physician participant, we used their ratings of all patients evaluated to determine the sensitivity and specificity of their assessment that a patient “appeared chronically ill” for the detection of poor health status. We examined two different criteria for the definition of poor health: SF-12 physical health score ≥1 SD or ≥2 SD below the age group norm. For the entire group of physicians, the median and inter-quartile range (IQR) for sensitivity, specificity, positive likelihood ratio, and negative likelihood ratio were calculated. The data were assumed to be sufficiently independent to utilize a T-test to compare the mean sensitivity and specificity between residents and faculty physicians. T-tests were also used to compare the mean sensitivity and specificity of physician assessments of patient participants with an age <50 years old to those ≥50 years old.

We also identified patients who were rated as “appearing chronically ill” by a majority of physicians, defined as greater than 75 percent of physician participants. We determined if patient characteristics were associated with being rated as “appearing chronically ill” by a majority of physicians using Chi-square tests (for gender, age, smoking status, and household income level) and Fisher’s exact test (for ethnicity). SPSS version 16 (SPSS, Inc., Chicago, IL) and SAS version 9.2 (SAS Institute Inc., Cary, NC) were used to perform statistical analyses.

## Results

Characteristics of the 126 patient participants are shown in Table 1. Mean age was 46.2 years (SD, 9.0) and the patients’ SF-12 physical composite scores were on average 5.3 points (SD, 12.0) below age-group norms. Forty-three patient participants (34%) were greater than or equal to 50 years of age. Sixty-two patient participants (49%) had one or more chronic conditions and a total of 42 (33%) had an SF-12 physical health score ≥1 SD below the age group norms and 22 patients (18%) had physical health scores that were ≥2 SD below the age-group norms.

**Table 1:** Characteristics of patient participants (n = 126 patients).

Characteristic		Number (%)
Sex	Male	63 (50)
Race	White	80 (65)
	Black	12 (10)
	Hispanic	8 (6)
	Other	26 (19)
Education	Not a high school graduate	20 (16)
	High school graduate	17 (14)
	Some university/college	33 (26)
	University/college degree	56 (44)
Annual HouseholdIncome	≤$20,000	45 (36)
	$20,001 to $60,000	35 (28)
	$60,001 to $100,000	21 (17)
	≥$100,000	25 (20)
Employment Status	Working full-time or part-time	63 (50)
	On disability	43 (34)
	Homemaker	5 (4)
	Student	3 (2)
	Unemployed	5 (4)
	Retired	7 (6)
Smoking Status	Daily or occasional	52 (41)
	Non-smoker	63 (50)
	No response	11 (9)
Count of ChronicConditions†	0	64 (51)
	1	37 (29)
	2	12 (10)
	≥3	13 (10)
Physical Health Score‡	≥1.0 SD (10 points) below norm	42 (33)
	≥2.0 SD (20 points) below norm	22 (18)

† Based on self-report, selecting from a list of eight specified conditions: (1) heart disease, such as angina, heart attack, or congestive heart failure (CHF); (2) lung disease, such as asthma, emphysema, chronic bronchitis, or chronic obstructive pulmonary disease (COPD); (3) liver disease, such as cirrhosis or chronic hepatitis; (4) intestinal or stomach ulcers, or other bowel disorders, such as Crohn’s disease or ulcerative colitis; (5) kidney failure or chronic kidney disease; (6) diabetes; (7) arthritis; and (8) stroke.

‡ Assessed by the SF-12 and classified using four different criteria based on number of standard deviations below the age-group norm (see text for details).

Of the 293 physicians invited to participate in the study, 117 (40%) responded. Six physicians rated less than 50% of images and were therefore excluded from the analysis. Of the 111 physician participants included in the analysis, 79 were internal medicine residents and 32 were general internal medicine faculty physicians.

Physicians rated a mean of 28% (range 2 to 55%) of patients as appearing chronically ill. Of the 126 patient participants, only eighteen were rated as appearing chronically ill by greater than 75% of physician participants. When poor health status was defined as an SF-12 physical score ≥1 SD below age group norms, the median sensitivity was 38.1%, median specificity 78.6%, median positive likelihood ratio 1.64, and median negative likelihood ratio 0.82. When poor health status was defined as an SF-12 physical score ≥2 SD below age group norms, the median sensitivity was 45.5%, median specificity 76.9%, median positive likelihood ratio 1.77, and median negative likelihood 0.75. These findings are shown in Table 2.

**Table 2:** Sensitivity, Specificity, Positive and Negative Likelihood Ratios of “Appearing Chronically Ill” for the Detection of Poor Health Status (n = 111 physicians).

	Appearing Chronically Ill			
	Sensitivity,median (IQR)^1^	Specificity,median (IQR)	Likelihood Ratio (+),median (IQR)	Likelihood Ratio (−),median (IQR)
SF-12 Physical Health Score≥1.0 SD below age-group norm	38.1 (28.6–47.6)	78.6 (69.0–84.0)	1.64 (1.42–2.15)	0.82 (0.74–0.87)
SF-12 Physical Health Score≥2.0 SD below age-group norm	45.5 (36.4–54.5)	76.9 (66.3–83.7)	1.77 (1.49–2.25)	0.75 (0.66–0.86)

1 IQR denotes inter-quartile range.

When faculty and resident physicians were compared, median sensitivity, specificity, and positive and negative likelihood ratios did not differ significantly (Table 3). When poor health status was defined as ≥2 SD below age-group norms, the specificity of “appearing chronically ill” was significantly greater for patients aged ≥50 years compared to those <50 years of age (p = 0.003). These findings are summarized in Table 4.

**Table 3:** Sensitivity and Specificity of “Appearing Chronically Ill” for the Detection of Poor Health Status, Resident Physicians Compared to Faculty Physicians.

	Appearing Chronically Ill			
	Resident Physicians*		Faculty Physicians	
	Sensitivity,median (IQR)	Specificity,median (IQR)	Specificity,median (IQR)	Specificity,median (IQR)
SF-12 Physical Health Score≥1.0 SD below age-group norm	38.1 (28.6–47.6)	77.4 (69.0–83.3)	36.9 (25.9–49.3)	81.6 (70.2–85.7)
SF-12 Physical Health Score≥2.0 SD below age-group norm	45.5 (36.4–54.5)	75.0 (66.3–83.7)	38.6 (31.8–54.1)	79.8 (68.5–84.6)

* When faculty and resident physicians were compared, median sensitivity, specificity, and positive and negative likelihood ratios did not differ significantly at p<0.05.

**Table 4:** Sensitivity and Specificity of “Appearing Chronically Ill” for the Detection of Poor Health Status, Patients Greater than 50 Years of Age compared to Patients Less Than 50 Years of Age.

	Appearing Chronically Ill			
	Greater than 50 years of age		Less than 50 years of age	
	Sensitivity,median (IQR)	Specificity,median (IQR)	Sensitivity,median (IQR)	Specificity,median (IQR)
SF-12 Physical Health Score≥1.0 SD below age-group norm	33.3 (27.8–50.0)	76.0 (64.0–84.0)	37.5 (29.2–45.8)	79.7 (71.2–86.4)
SF-12 Physical Health Score≥2.0 SD below age-group norm	33.3 (22.2–55.6)	73.5 (61.8–82.4)*	46.2 (38.5–61.5)	78.6 (68.6–85.7)

* When poor health status was defined as ≥2 SD below age-group norms, the specificity of “appearing chronically ill” was significantly greater for patients aged ≥50 years compared to those <50 years of age with p = 0.003.

Amongst those patients rated by a majority of physicians as “appearing chronically ill”, the only significantly associated patient characteristic was household income level (p = 0.004). Gender, age, smoking status and ethnicity were not found to be significantly associated with being perceived as chronically ill by the majority of physicians.

## Discussion

Physicians have long been taught that the physical examination is not complete without an assessment of a patient’s apparent health status. However, the findings of our study suggest that the diagnostic value of a physician’s assessment that a patient “appears chronically ill” is limited, with poor sensitivity and only modest specificity for the detection of poor health in adult outpatients. The median positive likelihood ratio was 1.49–2.45 when poor health status was defined as an SF-12 score ≥2 SD below age group norms, indicating that appearing chronically ill is associated with a small increase in the likelihood of poor health.

To the best of our knowledge, this is the first study to examine the sensitivity and specificity of “appearing chronically ill” for the detection of poor health status. In a related study by Gjørup et al in Denmark, four physicians and two medical students examined 201 adult in-patients and assessed whether the patient appeared acutely ill, chronically ill, or not ill [8]. The inter-rater reliability for “appearing chronically ill” varied considerably and ranged from 0.12 to 0.72 with wide confidence intervals. The authors hypothesized that the marked disagreement between raters was possibly due to the lack of an established definition of “appearing ill”. In a subsequent study by the same group, three physicians identified ten physical examination findings thought to be important in determining if a patient “appears ill”. The authors found that each physician used a different combination of physical examination findings to inform their clinical assessment of 201 inpatients, highlighting the absence of a uniform approach to determining a patient’s apparent health status [9]. Unlike other descriptors of a patient’s appearance such as jaundice or cyanosis, “appearing chronically ill” is difficult to characterize and definitions may vary between physicians. Our findings suggest that this may limit its utility as a component of the physical examination. Nonetheless, this study advances our understanding of the diagnostic value of a physician’s assessment that a patient “appears chronically ill” by determining the median sensitivity and specificity for a large number of physicians and employing the well-validated SF-12 as the reference standard.

Our study has certain limitations. First, the patients who participated in this study were recruited from an outpatient setting; thus, our findings may not be generalizable to the assessment of hospitalized patients who are acutely ill and likely have a higher prevalence of chronic disease. Second, the median age of participants was 46.2 (30 to 70) years. As such, the results of our study may not be applicable to patients outside of this range, in particular to patients greater than 70 years of age who may also have a greater burden of co-morbid disease. Third, only half of the patient participants in our study had at least one chronic condition, suggesting that the patients may be healthier than the general population. However, a greater number of patient participants had SF-12 scores ≥2 SD below age-group norms than would be expected (18%), suggesting that a significant proportion of patients had poor health status. Fourth, the response rate amongst physician participants was 40%. Although low, this response rate is typical for electronic surveys. Lastly, physicians assessed the apparent health status of patient participants using a digital photograph of the individuals’ face. A more robust assessment might be obtained in-person or through the review of videos of patients, as physicians are known to rely on multiple findings on physical examination to formulate their assessment of a patient’s apparent health status [9].

The study also suggests that clinical experience may not improve the sensitivity and specificity of a physician’s assessment that a patient “appears chronically ill”, as these values did not differ significantly between residents and faculty. In addition, the study found that the specificity of “appearing chronically ill” for the detection of poor health status was significantly higher in patients ≥50 years of age. This suggests that the assessment may offer greater discrimination in older patients. Future research in the area could aim to identify variables that influence a physician’s assessment of a patient’s apparent health status and determine whether this assessment offers any incremental value to the process of clinical decision-making.

Thus, our findings suggest that despite its established role in the physical examination, a physician’s assessment that a patient “appears chronically ill” has poor sensitivity and modest specificity for the detection of poor health status in adult outpatients. The likelihood ratios associated with “appearing chronically ill” indicate that this assessment has limited diagnostic value.

## Supporting Information

Figure S1 **Photographs of actual patient participants, as displayed to physician participants.**
(TIF)

Figure S2 **Photographs of actual patient participants, as displayed to physician participants.**
(TIF)

Figure S3 **Photographs of actual patient participants, as displayed to physician participants.**
(TIF)

Figure S4 **Photographs of actual patient participants, as displayed to physician participants.**
(TIF)

Document S1 **Patient consent forms.**
(PDF)
