# Supplementary figures and images for: Is “Appearing Chronically Ill” a Sign of Poor Health? A Study of Diagnostic Accuracy
Source: PLoS One. 2013 Nov 27;8(11):e79934. doi: 10.1371/journal.pone.0079934 (PMC3842283; doi:10.1371/journal.pone.0079934)

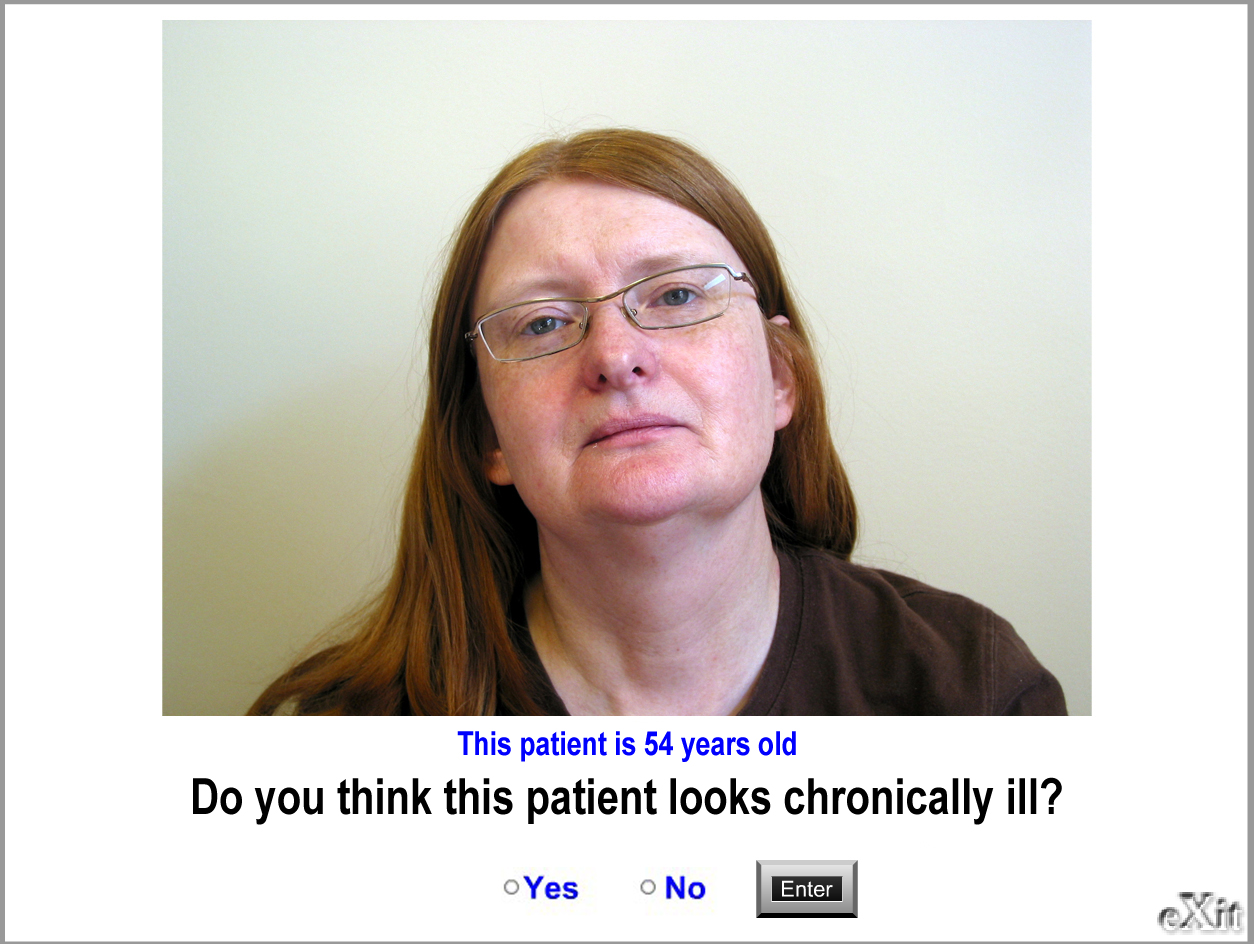

Supplement: Figure S1 — Photographs of actual patient participants, as displayed to physician participants. (TIF) [file pone.0079934.s001.tif]

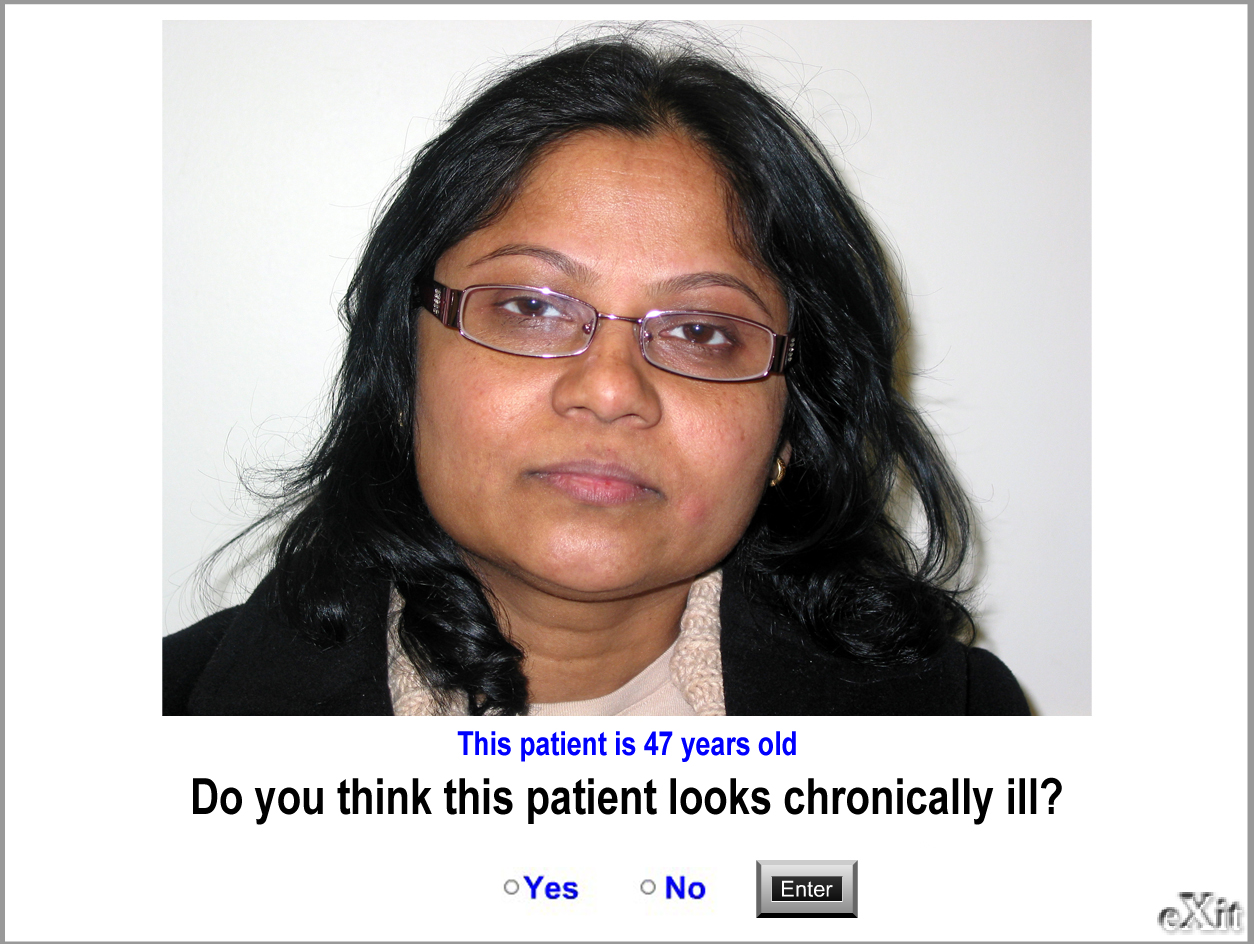

Supplement: Figure S2 — Photographs of actual patient participants, as displayed to physician participants. (TIF) [file pone.0079934.s002.tif]

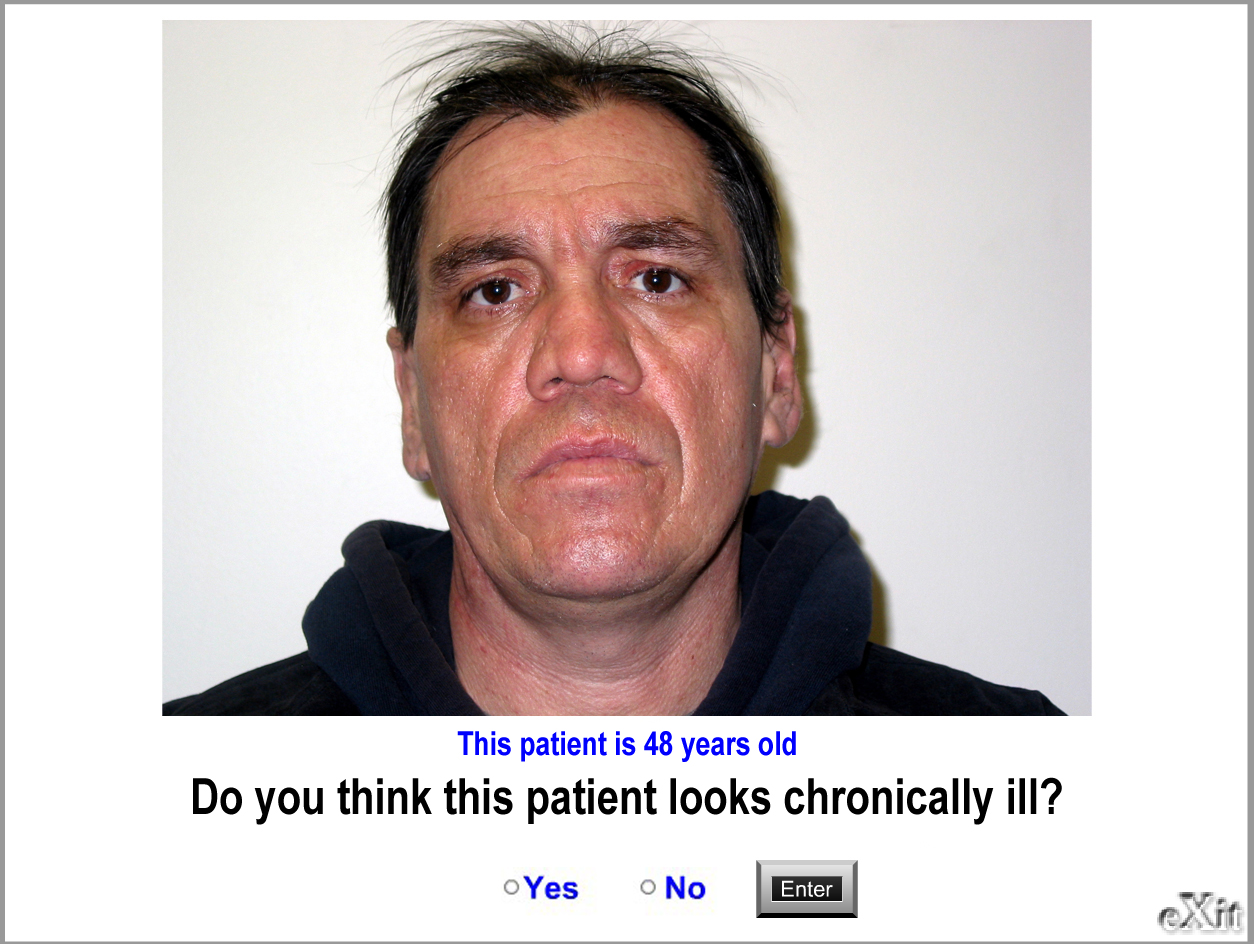

Supplement: Figure S3 — Photographs of actual patient participants, as displayed to physician participants. (TIF) [file pone.0079934.s003.tif]

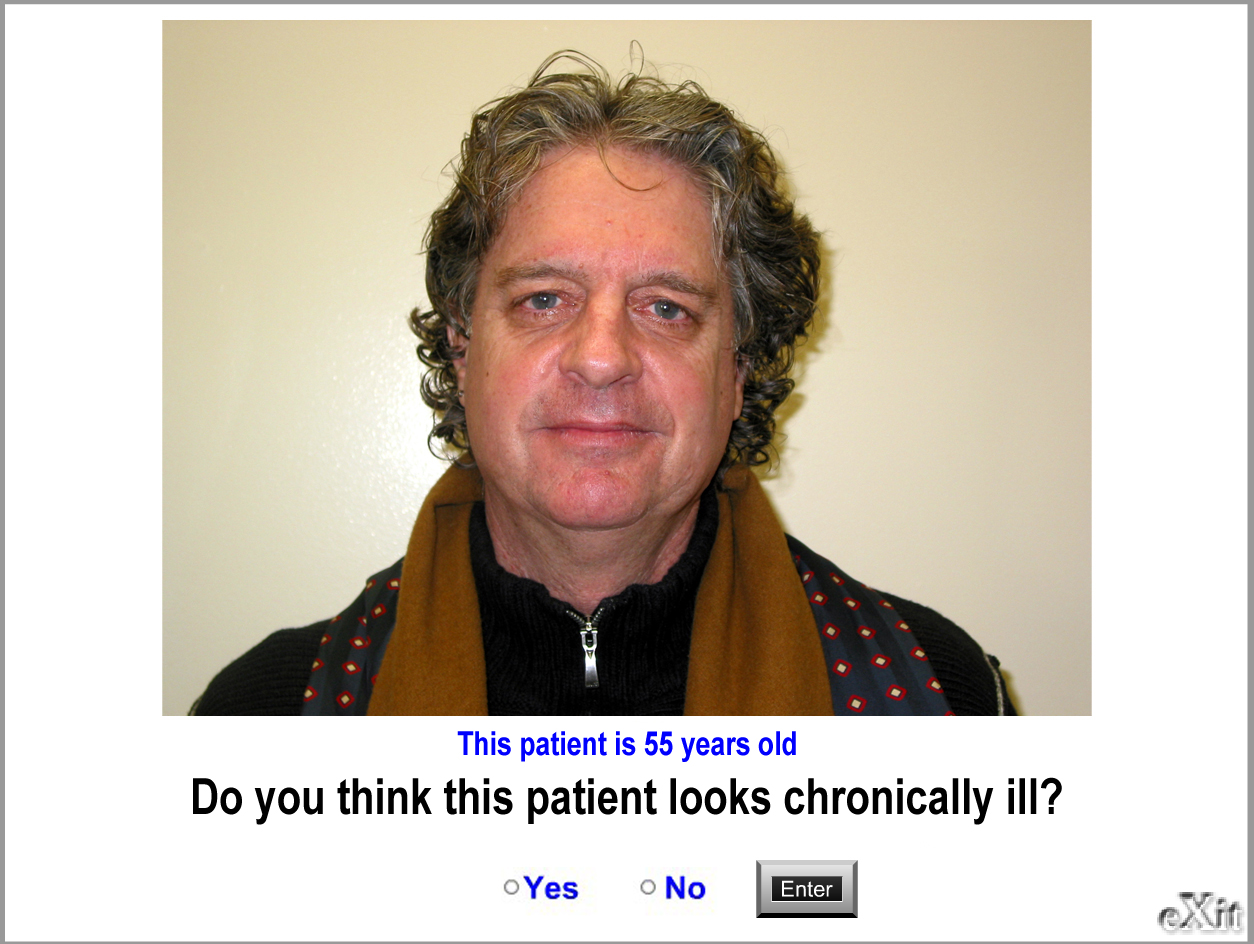

Supplement: Figure S4 — Photographs of actual patient participants, as displayed to physician participants. (TIF) [file pone.0079934.s004.tif]
