# Supplementary material for: Is “Appearing Chronically Ill” a Sign of Poor Health? A Study of Diagnostic Accuracy
Source: PLoS One. 2013 Nov 27;8(11):e79934. doi: 10.1371/journal.pone.0079934 (PMC3842283; doi:10.1371/journal.pone.0079934)
Supplement: Document S1 — Patient consent forms. (PDF) [file pone.0079934.s005.pdf]

St. Michael's

Inspired Care. Inspiring Science.

Consent Form Addendum

Project Title: The Relationship between Apparent Age, Health Status, and Socioeconomic Status

Researchers:
Dr. Stephen Hwang & Mina Atia
Centre for Research on Inner City Health
St. Michael's Hospital
30 Bond St. Toronto, ON M5B 1W8

Introduction

Before you sign this form, it is important that you read and understand the information given here regarding changes to the study you consented to participate in earlier. This portion of the study is voluntary and you may choose to not participate. If you have any questions, you can ask the study staff. You may also wish to discuss this with your family or friends or someone you trust.

Addendum to the Study

In early 2009, you participated in a research study called "The Relationship between Apparent Age, Health Status, and Socioeconomic Status". The goal of this study is to find out if physicians can tell if a patient is unwell or not just by looking at their photograph. For the study, we took your photograph, and you filled out a brief survey about your health.

We will soon be publishing the results of the study in a medical journal. The purpose of this letter is to ask your permission to publish your photograph along with your age in a medical journal. We have selected your photograph for publication because it is a good example of the photographs used in the study. Your photograph and your age at the time it was taken, as it would appear in the journal, are attached for you to review. Note that except for your age, we will not publish any information from the survey that you filled out.

Your consent to allow us to publish your photograph in a medical journal is completely voluntary. You will not be compensated in any way for this, and your decision to allow or not allow publication will not affect the care you receive at St. Michael's Hospital.

If you decide to consent to the publication of your photograph and age, you will be over-riding the following sections in the original consent form that you signed for the study:

> "All of the information you provide, including the survey and the photograph, will be kept strictly private and confidential."

> "In the event that the results of this study are published or presented at conferences, seminars or other public forums, no individual information or information that could identify you will be released."

Please note that we may choose to not publish your photograph, even if you give us permission to do so.

Research Ethics Board Contact Information:
If you have any questions about your rights as a research participant, you may contact The Chair, Research Ethics Board, St. Michael's Hospital, at 416-864-6060 Ext. 2557.

Study Contact Information
If you have any questions about this study, you may contact Dr. Stephen Hwang at the Centre for Research in Inner City Health at (416) 864-5991.

Project Title: The Relationship between Apparent Age, Health Status, and Socioeconomic Status

Consent Form Addendum
To be completed by Participants

I have read this addendum and I understand the research study and what is being asked of me.

I give permission for my photograph, along with my age, to be published in a medical journal.

J. D. W[illegible]	JOHN DOUGLAS WALLACE	SEPT. 6TH / 2010
**Participant Signature**	**Participant Name (please print)**	**Date**
[illegible]	Mina Atia	Aug 30 / 2010
**Signature of Investigator explaining study**	**Investigator Name (Printed)**	**Date**

Project Title: The Relationship between Apparent Age, Health Status, and Socioeconomic Status

Consent Form Addendum
To be completed by Participants

I have read this addendum and I understand the research study and what is being asked of me.

I give permission for my photograph, along with my age, to be published in a medical journal.

[signature]	Martha Kra	7 Sep 2010
**Participant Signature**	**Participant Name (please print)**	**Date**
[signature]	Mina Atia	Aug 30/2010
**Signature of Investigator explaining study**	**Investigator Name (Printed)**	**Date**

Version Date: August 25, 2010

Project Title: The Relationship between Apparent Age, Health Status, and Socioeconomic Status

Consent Form Addendum
To be completed by Participants

I have read this addendum and I understand the research study and what is being asked of me.

I give permission for my photograph, along with my age, to be published in a medical journal.

[signature]	PATRICK BURNS	SEPT. 2, 2010
**Participant Signature**	**Participant Name (please print)**	**Date**

[signature]	Mina Attia	Aug 30/2010
**Signature of Investigator explaining study**	**Investigator Name (Printed)**	**Date**

Version Date: August 25, 2010

Project Title: The Relationship between Apparent Age, Health Status, and Socioeconomic Status

Consent Form Addendum
To be completed by Participants

I have read this addendum and I understand the research study and what is being asked of me.

I give permission for my photograph, along with my age, to be published in a medical journal.

[illegible]	Niranjala Swam	Sept 9 2010
**Participant Signature**	**Participant Name (please print)**	**Date**
[illegible]	Mina Atia	Aug 30/2010
**Signature of Investigator explaining study**	**Investigator Name (Printed)**	**Date**
